# Pushing the limits of high-resolution polymer microscopy using antioxidants

**DOI:** 10.1038/s41467-020-20363-1

**Published:** 2021-01-08

**Authors:** Brooke Kuei, Enrique D. Gomez

**Affiliations:** 1 Department of Materials Science and Engineering, The Pennsylvania State University, University Park, Pennsylvania 16802 USA; 2 Department of Chemical Engineering, The Pennsylvania State University, University Park, Pennsylvania 16802 USA; 3 Materials Research Institute, The Pennsylvania State University, University Park, Pennsylvania 16802 USA

**Keywords:** Polymers, Imaging techniques, Transmission electron microscopy

## Abstract

High-resolution transmission electron microscopy (HRTEM) has been transformative to the field of polymer science, enabling the direct imaging of molecular structures. Although some materials have remarkable stability under electron beams, most HRTEM studies are limited by the electron dose the sample can handle. Beam damage of conjugated polymers is not yet fully understood, but it has been suggested that the diffusion of secondary reacting species may play a role. As such, we examine the effect of the addition of antioxidants to a series of solution-processable conjugated polymers as an approach to mitigating beam damage. Characterizing the effects of beam damage by calculating critical dose *D*_*C*_ values from the decay of electron diffraction peaks shows that beam damage of conjugated polymers in the TEM can be minimized by using antioxidants at room temperature, even if the antioxidant does not alter or incorporate into polymer crystals. As a consequence, the addition of antioxidants pushes the resolution limit of polymer microscopy, enabling imaging of a 3.6 Å lattice spacing in poly[(5,6-difluoro-2,1,3-benzothiadiazol-4,7-diyl)-alt-(3,3″′-di(2-octyldodecyl)-2,2′;5′,2″;5″,2″′-quaterthiophene-5,5″′-diyl)] (PffBT4T-2OD).

## Abstract (web summary)

High-resolution transmission electron microscopy (HRTEM) has been transformative to the field of polymer science but most HRTEM studies are limited by beam damage on the material. Here, the authors examine the effect of the addition of antioxidants to a series of conjugated polymers and demonstrate minimized beam damage at increased resolution.

## Introduction

Imaging with sub-nanometer resolution continues to be a challenge in high-resolution transmission electron microscopy (HRTEM) of polymers. A few examples have highlighted the potential of high-resolution polymer microscopy, such as the direct visualization of poly(*p*-phenylenebenzobisoxazole) (PBZO) chains packing at 3.5 Å that show local orientations of backbones and identifies defects within fibers^1^. Such alignment and defect population are important for the high modulus and strength of PBZO fibers. Imaging the (210) and (001) planes at 5.4 Å and 3.8 Å, respectively, was also important to reveal the twisting of crystals in poly(*m-*phenylene isophthalamide) (MPDI), demonstrating the possibility of helical structures from achiral molecules^2^. Sub-nanometer imaging of polymers, however, is only possible for a few systems.

Mapping chain packing in solution-processable conjugated polymers is crucial to identify the structural features responsible for charge conduction that is needed to enable a variety of organic optoelectronics^3–22^. Generally, these materials appear much more beam sensitive than the aforementioned examples, such that HRTEM has mostly focused on imaging lamellar alkyl stacking at about 15–20 Å^19,23,24^. HRTEM of poly(3-hexylthiophene-2,5-diyl) (P3HT), for example, reveals how P3HT crystals are ordered within fibrils^25^ and how molecular weight affects the extension of crystalline lamellae^26^. More recent studies have also looked at push-pull materials used in high-performance devices, revealing highly ordered lamellar nanostructures and relative orientation between adjacent domains in poly([N,N′-bis(2-octyldodecyl)-naphthalene-1,4,5,8-bis(dicarboximide)-2,6-diyl]-alt-5,5′-(2,2′-bithiophene)) (PNDI2OD-T2)^27^, and the effect of alkyl side chains on intercrystallite ordering in poly(benzo[1,2-b:4,5-b′]dithiophene−thieno[3,4-c]pyrrole-4,6-dione) (PBDTTPD)^28^. Despite progress from resolving the ~20 Å spacings of solution-processable conjugated polymers, imaging of the ~4 Å π-π stacking remains a challenge. This limitation, which has prevented the study of pathways for charge transport, is due to the inherent beam sensitivity of polymers^29–37^.

The primary damage process in organic materials is inelastic scattering, which causes molecular excitations or ionization. The energy dissipated either causes molecular vibrations (heat) or causes bond scission. In the case of bond scission, loss of hydrogen atoms and scission of carbon chains or side groups are followed by secondary processes that could significantly alter molecular structure^38^. For example, previous work has identified secondary processes as a possible contributor to radiation damage in polystyrene^39^, and we have hypothesized that diffusion of reacting species generated by side chain scission plays a significant role in beam damage of conjugated polymers^40,41^. Better damage mitigation strategies are needed to achieve sub-nanometer resolution.

Cryogenic conditions can reduce damage in the TEM^42^, enabling high resolution imaging by suppressing atomic motion even after bonds are broken, or by limiting the diffusion of secondary reacting species. Nevertheless, some soft materials are difficult to cool to cryogenic temperatures^43^ and cryogenic data can hide conformational diversity^44^. Several solid–solid phase transformations in organic semiconductors cannot be observed at cryogenic conditions^45^. As such, imaging at room temperature would be enabling for many experiments, and open the door for various in-situ studies.

In-situ heating in the TEM has offered valuable contributions to organic-inorganic systems, such as in monitoring degradation of organometallic halide perovskite solar cells under heating^46^ or in observing the loss of a capping polymer in the heating of polymer-capped platinum nanocrystals^47^. In-situ TEM electrochemistry enabled the electrochemical polymerization of poly(3,4-ethylenedioxythiophene) (PEDOT) to be visualized, revealing that particulates are formed in solution as the deposition proceeds, followed by deposition of these polymer particles; this refined previous descriptions of electrochemical polymerization^48^. Liquid TEM experiments of polymers include imaging changes in polymer conformations as a function of time in solution^49^ and investigating micelle fusion and growth in solution^50^. Finding approaches to minimize damage in the TEM at room temperature is crucial to continue to push the field of polymer microscopy forward.

Radical scavengers have been used to reduce the effects of irradiation in polyethylene^51^ and rubber^52^. In the biology community, radical scavengers have also been used to prevent radiation damage during small-angle X-ray scattering (SAXS) and crystallography experiments at room temperature. For example, styrene soaked into immunoglobulin crystals extend crystal lifetime^53^, uridine reduces damage in both SAXS and crystallography studies of lysosome^54^, sodium nitrate extends protein crystal lifetime of some proteins^55^, and free radical absorbers such as dithiothreitol (DTT) or ascorbate are suggested in solution SAXS protocols for the study of biological macromolecules^56^. Radical scavengers have also been used in liquid cell environments to quench radicals produced by the radiolysis of water molecules during microscopy experiments^57,58^. We propose that radical scavengers can be used to reduce damage of soft matter in the TEM at room temperature.

Here, we examine the effect of adding antioxidants on beam damage in a series of conjugated polymers. We chose the antioxidant butylated hydroxytoluene (BHT) because hindered phenols have been shown to stabilize organic solar cells from ultraviolet radiation damage^59^, but we also show suppression of beam damage with 2,2,6,6-Tetramethyl-1-piperidinyloxy (TEMPO). We show that the critical dose for damage (*D*_*C*_) increases with additives, indicating improved stability, even when the free radical scavenger does not incorporate into crystals. As a consequence, the addition of BHT enables HRTEM imaging of π-π stacking at 3.6 Å in poly[(5,6-difluoro-2,1,3-benzothiadiazol-4,7-diyl)-alt-(3,3″′-di(2-octyldodecyl)-2,2′;5′,2″;5″,2″′-quaterthiophene-5,5″′-diyl)] (PffBT4T-2OD) at room temperature, which is otherwise not possible with current instrumentation.

## Results

### Critical dose diffraction measurements

**Fig. 1:**
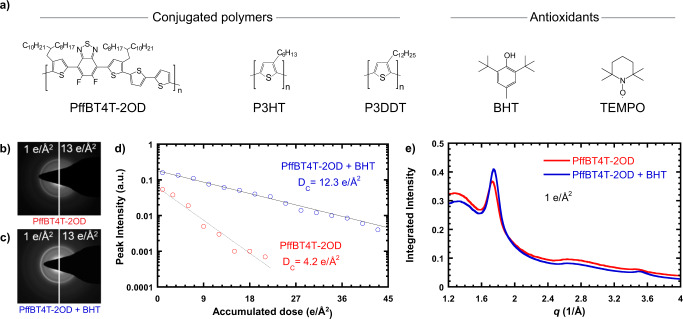
Chemical structures and minimization of beam damage by the addition of BHT at a dose rate of 1 e/Å^2^s at room temperature. (**a**) Chemical structures of conjugated polymers and antioxidants used in this study. (**b**) Electron diffraction pattern of neat PffBT4T-2OD at low and high dose, showing loss of crystal structure after an accumulated dose of 13 e/Å^2^. (**c**) Electron diffraction pattern of PffBT4T-2OD + BHT at low and high dose, showing partially preserved crystal structure at 13 e/Å^2^. (**d**) Background subtracted peak intensity vs. accumulated dose for PffBT4T-2OD and PffBT4T-2OD + BHT with exponential fits showing persistence of crystal structure to higher dose with the addition of BHT. (**e**) Azimuthal integration of electron diffraction patterns of PffBT4T-2OD with and without BHT at a dose of 1 e/Å^2^, showing no change in crystal structure.

Damage was characterized in a series of conjugated polymers with and without antioxidants to investigate the ability of antioxidants to reduce damage (chemical structures of polymers and antioxidants are shown in Fig. 1a). To quantitatively characterize beam damage in conjugated polymers, we calculated critical dose (*D*_*C*_) values from the decay of electron diffraction intensities. A series of electron diffraction patterns were collected at a dose rate of 1 e/Å^2^s. As shown in Fig. 1b, c, the π-π diffraction ring fades away (the semicrystalline structure is damaged) as dose is accumulated. Each diffraction pattern is azimuthally integrated (Supplementary Fig. 1, Supplementary Note 1) and the intensity of the π-π peak is plotted as a function of accumulated dose (Fig. 1d, Supplementary Fig. 2, Supplementary Note 2), revealing an exponential decay of the diffraction intensity. *D*_*C*_, which is taken as the electron dose at which the diffraction intensity drops to 1/*e* of its initial value (the dose above which a material is significantly changed), can be calculated by taking the inverse of the decay rate, as described by Eq. (1)

1 $$I = A\,{\mathrm{exp}}\left( { - \frac{D}{{D_c}}} \right) + I_b$$

where *I* is the diffraction peak intensity, *A* is a constant, *D* is the accumulated dose, and *I*_*b*_ is the background intensity^29^. *D*_*C*_ is a convenient convention, although others have characterized radiation sensitivity as the dose at which signal decreases to half its initial value or the dose at which signal is completely lost^60^. For PffBT4T-2OD, *D*_*C*_ is 4.2 e/Å^2^, and for PffBT4T-2OD + BHT *D*_*C*_ is 12.3 e/Å^2^. As such, at the critical dose for PffBT4T-2OD + BHT, the diffraction intensity is an order of magnitude higher than without BHT. As seen in Fig. 1e, Supplementary Fig. 3 and Supplementary Note 3, the addition of BHT does not change the diffraction patterns from PffBT4T-2OD, suggesting that the crystal structure is unperturbed. This implies that BHT resides in the amorphous areas of the sample and minimizes damage without altering the crystals.

**Fig. 2:**
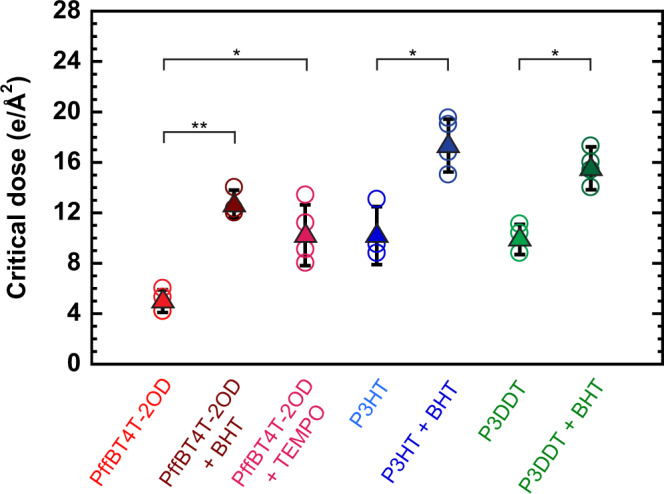
Critical doses of PffBT4T-2OD, P3HT, and P3DDT with and without antioxidant at room temperature. The addition of 7 wt% antioxidant increases the critical dose of conjugated polymers at 1 e/Å^2^s. BHT is most effective at minimizing damage in PffBT4T-2OD. BHT appears to be more effective at minimizing damage than TEMPO. Average values are shown as filled triangles and individual data points are shown as empty circles. Error bars are the standard deviation of multiple measurements (*n* ~ 3). Two sample, one-tailed p-values are shown as *p* < 0.05 (∗) and *p* < 0.01 (∗∗).

We show that the mitigation of beam damage by BHT is a general result by measuring the *D*_*C*_ of two additional conjugated polymers, poly(3-hexylthiophene-2,5-diyl) (P3HT) and poly(3-dodecylthiophene-2,5-diyl) (P3DDT), with and without BHT (examples of peak intensity vs. dose are shown in Supplementary Fig. 4). We find that the addition of BHT increases *D*_*C*_ for all three polymers (Fig. 2). We also show other antioxidants can stabilize polymer crystals in the TEM by adding a second antioxidant, TEMPO, to PffBT4T-2OD. Again, *D*_*C*_ increases with the addition of the free radical scavenger. The increase in *D*_*C*_ ranges from a factor of 1.5 for P3DDT to a factor of 3 for PffBT4T-2OD, indicating that diffraction signals are often an order of magnitude higher at doses near *D*_*C*_ when antioxidants are included (see Fig. 1d, Supplementary Fig. 4 and Supplementary Note 4).

### High-resolution TEM imaging with antioxidants

**Fig. 3:**
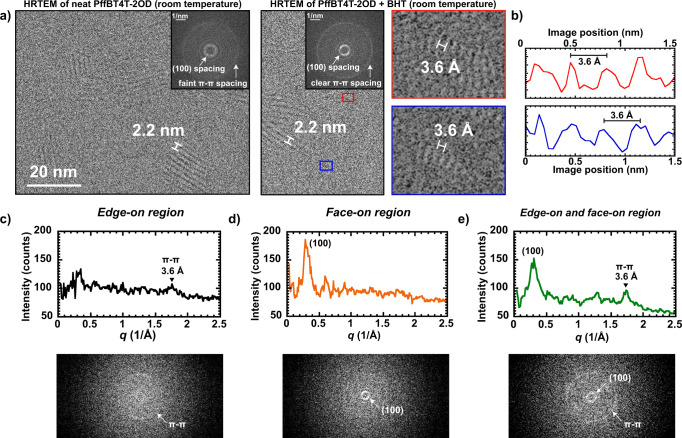
HRTEM at room temperature of PffBT4T-2OD without and with the addition of BHT. Without BHT, the 2.2 nm (100) lattice fringes can be seen, but π–π stacking remains elusive. With the addition of BHT, a clear π-π stacking ring is visible in the FFT, and Fourier filtering the image reveals 3.6 Å π–π stacks. Dose of HRTEM images is 10 e/Å^2^.

Increasing *D*_*C*_ should enable higher-resolution imaging. Without BHT, PffBT4T-2OD has a very low *D*_*C*_ of 4.2 e/Å^2^, suggesting that HRTEM with sub-nanometer resolution is not possible with current instrumentation, according to Glaeser’s limits of image formation that relate resolvable feature size to dose^31,61^. As shown in Fig. 3a, HRTEM of neat PffBT4T-2OD shows the 22 Å (100) lattice fringes that state-of-the-art TEM has been able to achieve. We can look at the fast Fourier transform (FFT) (see inset) for a representation of the image in reciprocal space, where bright spots or arcs correspond to diffraction and additional thon rings are from defocus. In the FFT from HRTEM of neat PffBT4T-2OD, diffraction spots from the (100) spacing are visible, but there is only a faint trace of a π-π stacking ring. On the other hand, in the FFT of HRTEM images of PffBT4T-2OD + BHT we see the (100) spacing and a clear ring at 2.78 1/nm (3.6 Å) that we attribute to evidence of π-π stacking. We Fourier filter the image to enhance structures contributing to the π-π stacking ring, as commonly done in polymer microscopy^26,30^, revealing the π stacks in real space as shown in the red and blue insets (additional images are shown in Supplementary Fig. 5, see also Supplementary Note 5). Line scans of π-π stacking regions from the original, unfiltered image demonstrate a periodicity corresponding to 3.6 Å, which is in agreement with π-π stacking distances from both electron diffraction and GIWAXS of PffBT4T-2OD (Fig. 3b)^15,62^. Thus, with the addition of BHT, we can image the 3.6 Å π-π spacing in a solution processed conjugated polymer at room temperature, which is otherwise not possible.

To confirm our assignment of the ring at 2.78 1/nm in the FFT to evidence of π-π stacking, we take the FFTs from various regions of interest (Supplementary Fig. 6a, Supplementary Note 6). We observe that in areas away from visible (100) spacings (Fig. 3c) a ring corresponding to π-π stacking is apparent in the FFT and its azimuthal integration, suggesting the presence of edge-on crystals in that region. In regions with visible (100) spacings in the image (Fig. 3d), only a (100) ring is apparent in FFTs, as expected for regions containing face-on crystals. In a region containing both visible (100) fringes and the area adjacent to it, we see features corresponding to both (100) planes and 3.6 Å stacking (Fig. 3e).

We can examine the effect of BHT more closely by looking at HRTEM images and FFTs of PffBT4T-2OD and PffBT4T-2OD + BHT as a function of dose (Supplementary Fig. 6b, c, Supplementary Note 6). In HRTEM of PffBT4T-2OD, the 22 Å (100) lattice fringes of PffBT4T-2OD are visible initially, but have faded after exposure to higher dose. On the other hand, in the case of PffBT4T-2OD + BHT, some (100) lattice fringes remain even at a high electron dose. This effect can be seen even more clearly in the FFTs, where the (100) diffraction spots or arcs are visible in the FFTs at 10 e/Å^2^ with and without BHT. By 70 e/Å^2^, these diffraction spots have faded away in the neat polymer but persist in the sample with BHT, demonstrating the ability of BHT to reduce beam damage at room temperature. As mentioned previously, a clear π-π ring is visible in the FFT with added BHT. The π-π ring is strong at 10 e/Å^2^ and persists at 20 e/Å^2^, whereas in the neat polymer, the ring is barely visible at 10 e/Å^2^ and has completely faded away by 20 e/Å^2^.

**Fig. 4:**
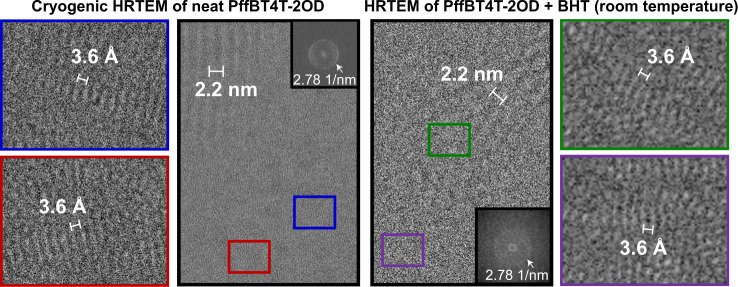
Comparison of π stacking morphology of neat PffBT4T-2OD at cryogenic conditions (dose: 80 e/Å^2^) vs. PffBT4T-2OD + BHT at room temperature (dose: 10 e/Å^2^). Main panels show the 2.2 nm lamellar stacking and insets show π-π stacking. Red and blue insets are unfiltered, while green and purple insets from room temperature imaging are Fourier-filtered. We observe the same morphology at room temperature with the addition of antioxidant as we do at cryogenic conditions, suggesting that antioxidants can be used for high-resolution room temperature experiments.

Because cryogenic imaging is a commonly used strategy for beam damage mitigation, we use this as a benchmark for this work. Although the signal-to-noise ratio is superior at cryogenic conditions (Supplementary Fig. 7, Supplementary Note 7), the π stacking morphology observed at cryogenic conditions is the same as what is observed at room temperature with the addition of BHT (Fig. 4). Thus, by minimizing beam damage through the addition of antioxidants, we have gained the ability to observe π-π stacking of a conjugated polymer thin-film at room temperature, which is not possible in the neat polymer.

## Discussion

We hypothesize that exposure to the electron beam generates free radical reacting species in the conjugated polymer (likely from scission within side chains) that then diffuse around, causing further damage to the material, potentially in a cascading manner (Supplementary Fig. 8, Supplementary Note 8)^41^. Thus, the addition of an antioxidant quenches reacting species before they cause further damage. BHT is more effective at minimizing damage in PffBT4T-2OD and P3HT than it is in P3DDT, which could be due to the higher fraction of side chains in P3DDT creating a higher population of reacting species. BHT is also more effective than TEMPO, likely because the BHT radical is more stable than a nitroxide radical (Supplementary Fig. 9, Supplementary Note 9). This supports the theory that damage occurs through the diffusion of a free radical reacting species and that the antioxidant helps by quenching these reacting species. It is likely that the radical being quenched is an alkyl radical, as this is commonly produced during irradiation of linear polymers such as polyethylene^63,64^. Antioxidants similar to BHT have been shown to quench alkyl radicals in irradiated polyethylene.^51^ As such, it is reasonable to predict that this method of mitigating electron beam damage in the TEM can be extended to other polymers besides conjugated polymers. Furthermore, we speculate that the use of radical scavengers could have two benefits in liquid cell TEM experiments of soft matter, where the scavenger can quench both radicals formed by radiolysis of water molecules and radicals formed by damage to the soft material.

Previous work has shown clear changes to diffraction patterns when small molecules enter crystalline polymer phases^65,66^. Figure 1e shows little or no change to the electron diffraction pattern of PffBT4T-2OD with the addition of BHT. This was also confirmed with grazing incidence wide angle X-ray scattering, which showed no difference in either in-plane or out-of-plane diffraction for all three polymers with and without BHT (Supplementary Fig. 3). Because we find no clear evidence of perturbations to the crystal structure of the polymer with the addition of an antioxidant, we speculate that the antioxidant resides in amorphous regions and is therefore able to stabilize polymer crystals from a distance. Damage likely occurs in the amorphous phase in ways that are not captured by electron diffraction damage experiments; further investigations into damage in the amorphous areas might help elucidate the damage mechanism in more detail.

In conclusion, we have demonstrated through both studies of the critical dose for damage and HRTEM imaging experiments that antioxidants can be used to minimize beam damage in conjugated polymers at room temperature, even if the antioxidant does not incorporate into polymer crystals. We hypothesize that diffusion of reacting species is a significant contributor to beam damage of conjugated polymers and that the addition of an antioxidant is able to minimize damage by quenching free radicals. As a consequence, we can use antioxidants to image π-π stacking at room temperature in a solution-processable conjugated polymer, which was not possible in the neat polymer. This work not only helps to shed light on the mechanism for beam damage in conjugated polymers, but also demonstrates an approach to minimize damage in the TEM, which can enable further high-resolution studies as well as in-situ experiments.

## Methods

For diffraction experiments, 10 mg/mL solutions of PffBT4T-2OD (8.8 kg/mol, Ð of 1.068, Solarmer), P3HT (50.9 kg/mol, Ð of 2.23, 96% H-T regioregularity, Merck), and P3DDT (60.0 kg/mol, regioregular, Sigma-Aldrich) were made with chlorobenzene (Sigma-Aldrich) in a nitrogen glove box and stirred for a minimum of 10 h at 45 °C. For imaging experiments, the concentration was reduced to 3 mg/mL for thinner samples to avoid imaging overlapping crystals. For samples containing BHT or TEMPO, the antioxidant was added in 7% concentration of solids. This concentration was found to be optimal (Supplementary Fig. 10, Supplementary Note 10) and is in agreement with the range used to mitigate UV radiation damage in organic photovoltaics^59^. Silicon wafers were cleaned through sonication for 20 min in acetone and 20 min in isopropanol followed by 15 min of ultraviolet light ozonation. PEDOT:PSS (Clevios P, H.C. Starck) was spin-coated onto the silicon wafers in air, after which the polymer of interest was spin-coated onto the PEDOT:PSS film inside a nitrogen glove box. Films were floated off in deionized water and then picked up with copper TEM grids. Samples were dried overnight at room temperature under vacuum and then annealed in a nitrogen glove box. P3HT samples were annealed at 165 °C for 3 h and P3DDT and PffBT4T-2OD samples were annealed at 130 °C for 1 h.

Diffraction experiments were carried out on the FEI Tecnai G20 XTWIN at the Penn State Materials Characterization Lab operating at 200 kV at room temperature with a LaB6 gun and UltraScan camera. This accelerating voltage is chosen to reduce ionization damage (80 kV would cause more damage than 200–300 kV^67^). Dose rates were measured in areas of vacuum in the sample, after which a selected area aperture was inserted, the beam was blanked, and a fresh location on the sample was moved to. There, the beam was unblanked and diffraction patterns were collected on the sample with 1 s exposure times at 3 s intervals using the Digital Micrograph Acquire Series plug-in. The sample was exposed to the beam during the 3 s intervals (not just during the exposures). A dose rate of 1 e/Å^2^s was used for all critical dose experiments. A camera length of 330 mm, defocus of 0, spot size 3, and pixel size of 0.013 1/nm were used.

Room temperature imaging experiments were carried out on the double-aberration-corrected TEAM I at the National Center for Electron Microscopy, Lawrence Berkeley National Lab operating at 300 kV at room temperature. Images were collected using a K2 direct electron detector in counted mode at a dose rate of 5 e/Å^2^s with 1 s frames accumulating up to a 70 s exposure stack (dose rate was chosen to optimize detector because too high of a dose rate complicates the ability of the detector to identify individual electron events but too low a dose rate makes it difficult to align frames with low counts). A magnification of 119kx, pixel size of 0.042 nm, and defocus of −3.00 µm were used. Images reported in this paper are aligned slices of the stack containing 10 e/Å^2^ each (2 frames). Inverse FFTs were calculated in Digital Micrograph. Fourier filtered insets in Fig. 3 are raw images overlaid with a 75% transparency inverse FFT of the data at 2.78 1/nm. Fourier filtered insets in Fig. 4 are raw images overlaid with a 50% transparency inverse FFT of the data at 2.78 1/nm.

High-resolution cryogenic imaging experiments were conducted on the Titan Krios at the Penn State Materials Characterization Lab operating at 300 kV with a Falcon 3ec direct electron detector in counted mode but without dose fractionation (due to a short exposure time). Grids with experimental samples were cooled to liquid nitrogen temperature inside the autoloader. The microscope was operated in nanoprobe mode with a spot size of 5 and an illuminated area of 0.65 µm at a magnification of 470,000x. We used a dose rate of 75 e/Å^2^s and an exposure time of 1.07 s. We used an applied defocus of −1.00 µm. No camera pixel binning was used (binning of 1).

## Supplementary information

Supplementary Information

## Data Availability

The data that supports the findings of this manuscript can be found in the Supplementary Information and on ScholarSphere, Penn State at https://scholarsphere.psu.edu/collections/ksn009z45d.

## Code availability

The custom Mathematica code used for azimuthal integration of diffraction patterns can be found on ScholarSphere, Penn State at https://scholarsphere.psu.edu/collections/ksn009z45d.
