# Supplementary material for: Pushing the limits of high-resolution polymer microscopy using antioxidants
Source: Nat Commun. 2021 Jan 8;12:153. doi: 10.1038/s41467-020-20363-1 (PMC7794589; doi:10.1038/s41467-020-20363-1)
Supplement: Supplementary file 1 — Supplementary Information [file 41467_2020_20363_MOESM1_ESM.pdf]

Supplementary Information

Pushing the limits of high-resolution polymer microscopy using antioxidants

Brooke Kuei[1] and Enrique D. Gomez[1,2,3]*

[1]Department of Materials Science and Engineering, The Pennsylvania State University, University Park, Pennsylvania 16802, United States
[2]Department of Chemical Engineering, The Pennsylvania State University, University Park, Pennsylvania 16802, United States
[3]Materials Research Institute, The Pennsylvania State University, University Park, Pennsylvania 16802, United States

*Email: edg12@psu.edu

Supplementary Figures

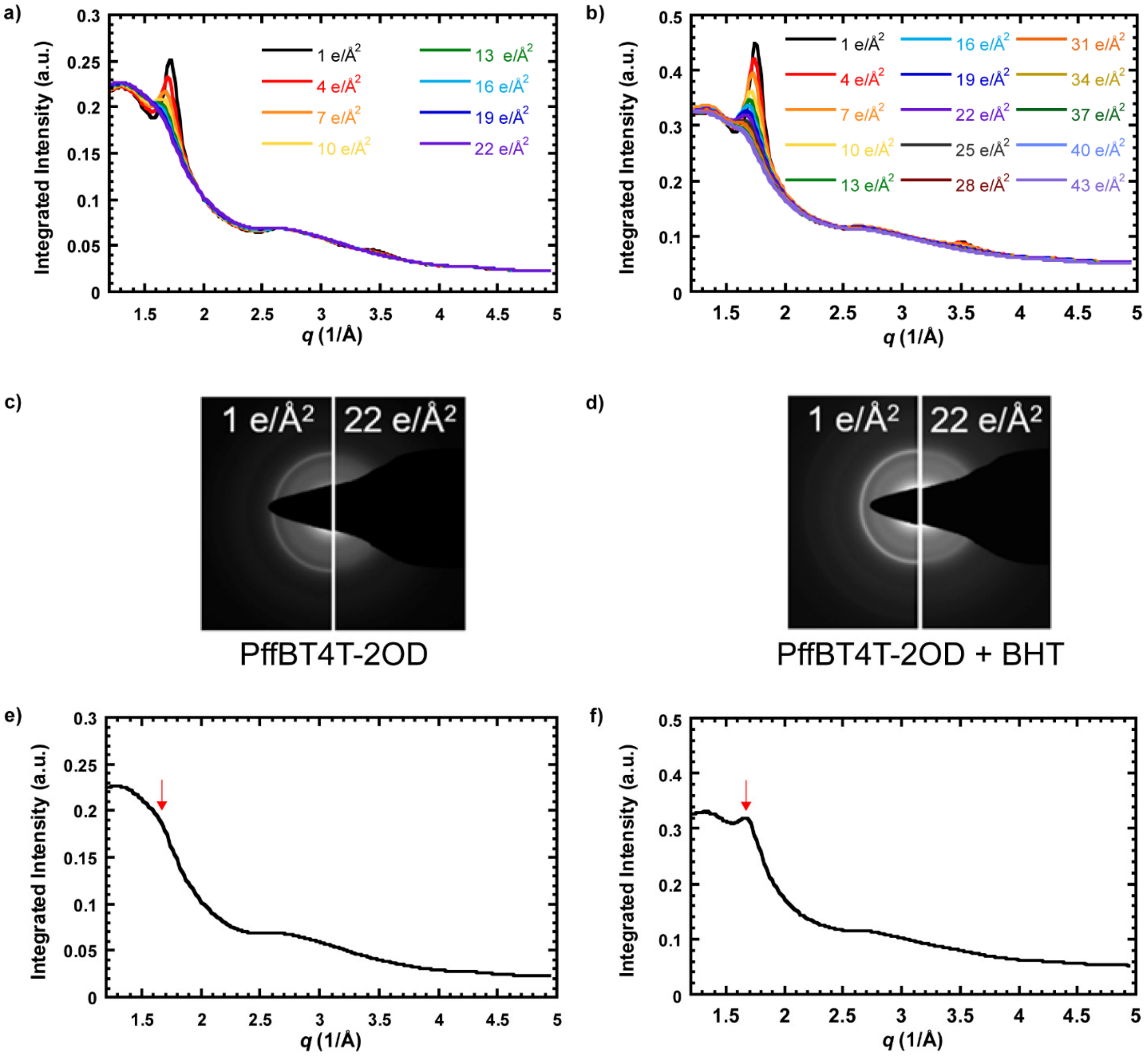

Supplementary Figure 1. (a) Azimuthal integration of diffraction rings from Fig. 1a. (b) Azimuthal integration of diffraction rings from Fig. 1b. (c) Electron diffraction patterns of PffBT4T-2OD at low and high dose, showing loss of crystal structure by 22 e/Å^2. (d) Electron diffraction patterns of PffBT4T-2OD + BHT at low and high dose, showing some preserved crystallinity by 22 e/Å^2. (e) Azimuthal integration of PffBT4T-2OD diffraction pattern at 22 e/Å^2, showing loss of π-π peak (indicated by red arrow). (f) Azimuthal integration of PffBT4T-2OD + BHT diffraction pattern at 22 e/Å^2, showing a partial π-π peak (indicated by red arrow).

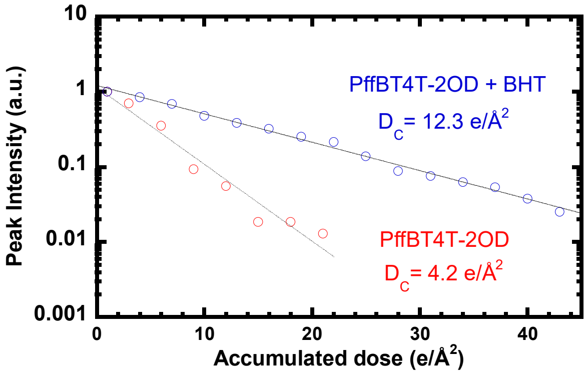

Supplementary Figure 2. Normalized peak intensity vs. accumulated dose for PffBT4T-2OD and PffBT4T-2OD + BHT.

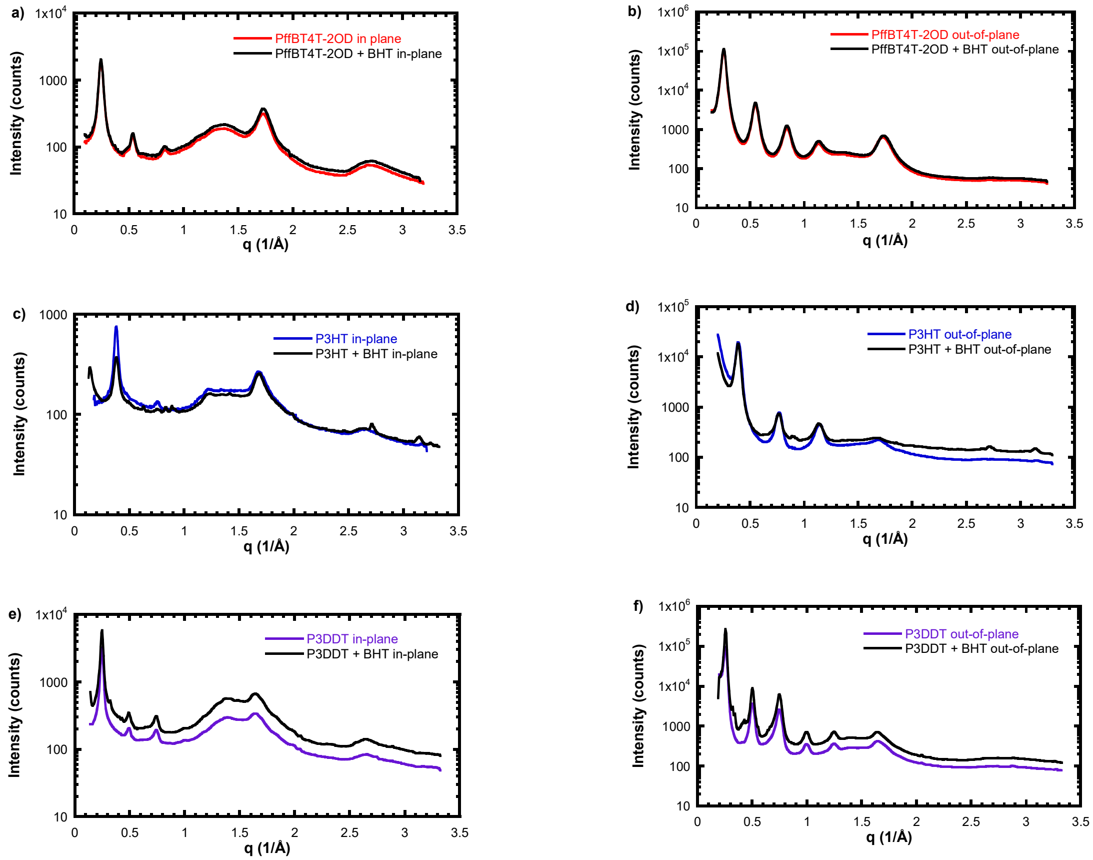

Supplementary Figure 3. Grazing incidence wide angle X-ray scattering of polymers with and without BHT demonstrating that the addition of an antioxidant does not alter the crystal structure of the polymer. PffBT4T-2OD and PffBT4T-2OD + BHT have identical (a) in-plane structure and (b) out-of-plane structure. P3HT and P3HT + BHT have identical (c) in-plane structure and (d) out-of-plane structure. P3DDT and P3DDT + BHT have identical (e) in-plane structure and (f) out-of-plane structure.

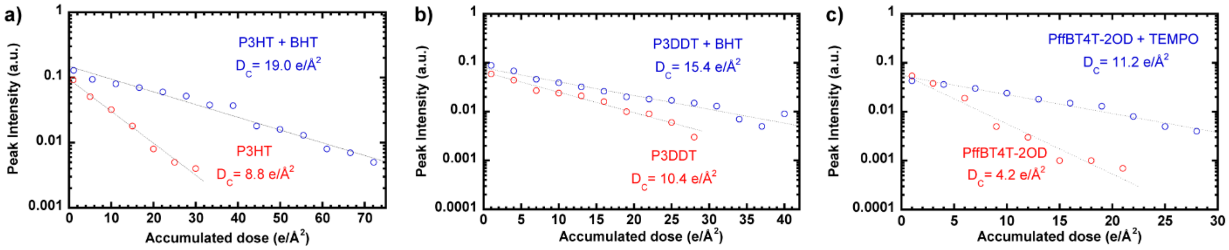

Supplementary Figure 4. Peak intensity vs dose for (a) P3HT without and with BHT, (b) P3DDT without and with BHT, and (c) PffBT4T-2OD without and with TEMPO.

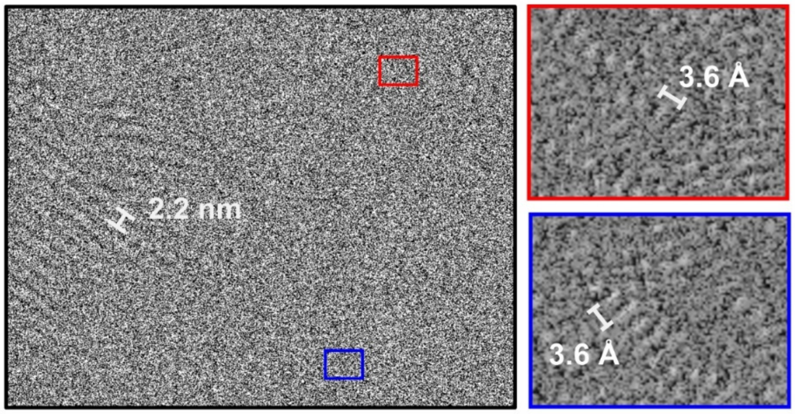

Supplementary Figure 5. Additional images showing π-π stacking in PffBT4T-2OD + BHT at room temperature. Insets are Fourier filtered by overlaying raw images with a 70% transparency inverse FFT of the data at 2.78 1/nm.

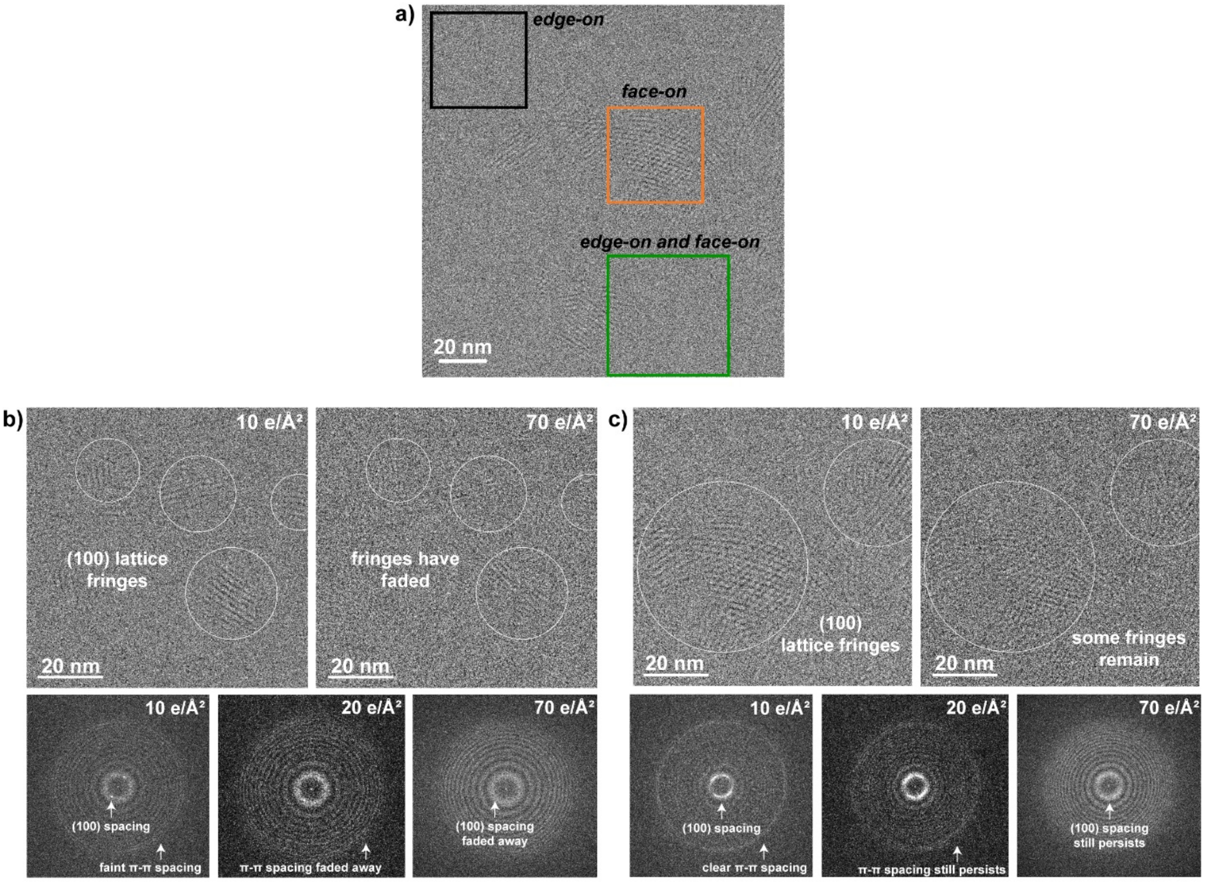

Supplementary Figure 6. Analysis of HRTEM images. (a) Regions of interest corresponding to edge-on, face-on, and edge-on and face-on areas where FFTs are taken in HRTEM of PffBT4T-2OD + BHT. (b,c) HRTEM images (not summed, each image containing 10 e/Å^2) and corresponding FFTs of PffB4T-2OD with and without BHT at 10 e/Å^2 and 70 e/Å^2. At 10 e/Å^2, the (100) diffraction spots corresponding to 22 Å spacings are visible in the FFTs with and without BHT. By 70 e/Å^2, these diffraction spots have faded away in the neat polymer sample but persist in the sample with BHT. While not visible in the images, the π-π stacking is visible in the FFT of PffBT4T-2OD + BHT at 10 e/Å^2 and still persists at 20 e/Å^2. In the FFT of PffBT4T-2OD, the π-π stacking is faint at 10 e/Å^2 and has faded away altogether by 20 e/Å^2. Images were acquired at a dose rate of 5 e/Å^{2}s (to optimize detector conditions) in counting mode on a K2 direct electron detector.

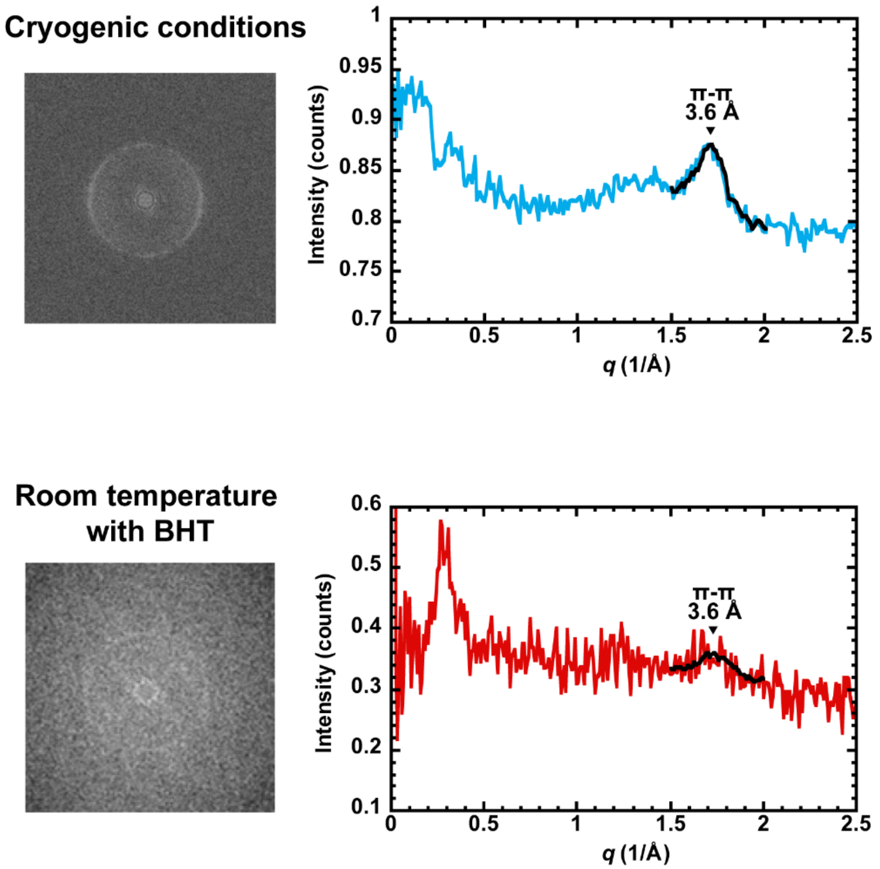

Supplementary Figure 7. FFTs and their radial integrations of HRTEM images of PffBT4T-2OD at cryogenic conditions vs PffBT4T-2OD + BHT at room temperature.

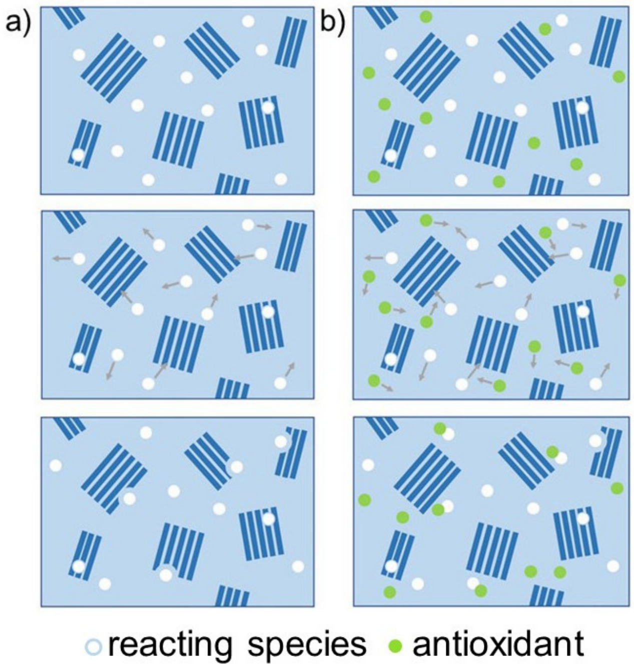

Supplementary Figure 8. Illustration of beam damage without and with antioxidant. Crystalline regions are dark blue and amorphous regions are light blue. (a) Without the addition of antioxidant, reacting species diffuse around and cause damage to crystalline regions. (b) With antioxidant, reacting species are quenched by the antioxidant before causing further damage.

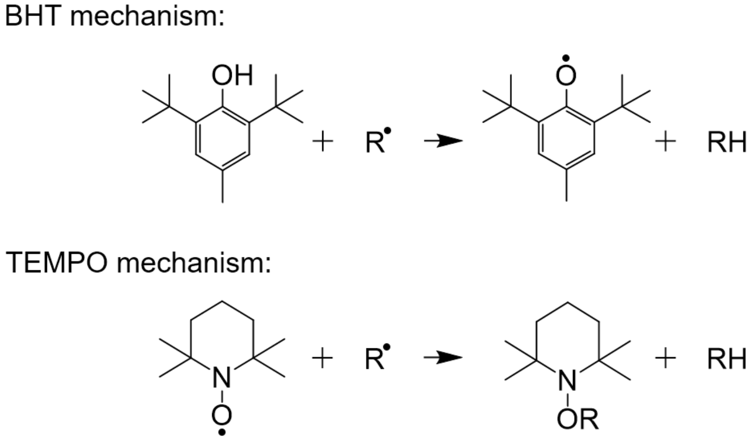

Supplementary Figure 9. Proposed mechanisms for how BHT and TEMPO quench reacting species.

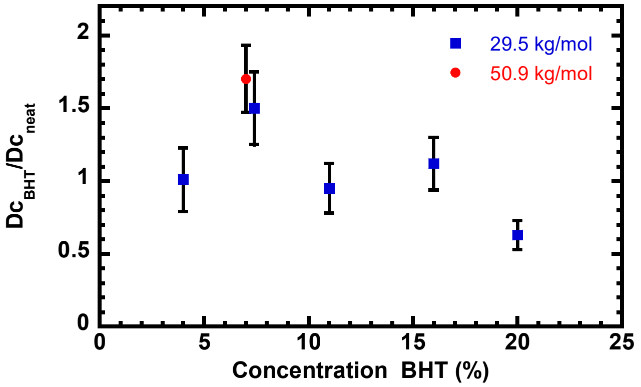

Supplementary Figure 10. *Dc_{BHT}/Dc_{neat}* vs concentration BHT for P3HT. Data for two molecular weights of P3HT is shown. At low concentrations, the addition of BHT has no effect, while at high concentrations, the addition of BHT leads to a lower critical dose. Critical dose is maximized with an addition of 7wt% BHT.

Supplementary Note 1

Radial integration of electron diffraction patterns. To quantitatively analyze our electron diffraction patterns, each pattern was azimuthally integrated with custom Mathematica code. **Supplementary Fig. 1a** shows the azimuthal integrations for PffBT4T-2OD at increasing accumulated dose and **Supplementary Fig. 1b** shows the azimuthal integrations of PffBT4T-2OD + BHT at increasing accumulated dose. For this study, we monitored the π-π peak at q = 1.72 1/Å, which can be seen decreasing in intensity with increasing accumulated dose. There is also a shift to lower q with increasing dose, indicating an increase in lattice spacing with damage. This is consistent with observations in other polymers[1, 2] and is likely due to decreased molecular interactions with damage, allowing chains to drift away from each other. The addition of BHT allows the π-π peak to persist to higher dose. **Supplementary Fig. 1c** and **Supplementary Fig. 1d** show diffraction patterns associated with neat PffBT4T-2OD and PffBT4T-2OD + BHT, respectively, at 1 e/Å^2 and at 22 e/Å^2. By a dose of 22 e/Å^2, PffBT4T-2OD has lost its diffraction

peak entirely (**Supplementary Fig. 1e**) whereas PffBT4T-2OD + BHT still shows some crystallinity (**Supplementary Fig. 1f**).

Supplementary Note 2

Normalized peak intensity vs. dose. To account for variations in diffraction intensity due to sample variability, **Supplementary Fig. 2** shows intensities from Fig. 1d normalized to the intensity near zero dose (the normalization has no effect on critical dose values).

Supplementary Note 3

Grazing incidence wide-angle X-ray scattering of polymer films with and without BHT. To confirm that the addition of BHT does not change the structure of the conjugated polymers, we performed grazing incidence wide-angle X-ray scattering on PffBT4T-2OD, P3HT, and P3DDT with and without BHT (**Supplementary Fig. 3**). The left-hand column shows the 1D in-plane data corresponding to horizontal line cuts and the right-hand column shows the 1D out-of-plane data corresponding to vertical line cuts. For all three polymers, both the in-plane and out-of-plane structure stay the same with the addition of BHT.

Supplementary Note 4

Peak intensity vs. dose without and with antioxidant. **Supplementary Fig. 4** shows similar data as Figure 1d with other polymers and antioxidants. In all cases, the addition of antioxidant alters the decay rate and enables preservation of the crystals to higher electron doses.

Supplementary Note 5

Additional room temperature TEM images showing π-π stacking. **Supplementary Fig. 5** shows additional examples of π-π stacking from the region shown in Fig. 3. With the addition of BHT, we gain the ability to see π stacks in a thin-film of PffBT4T-2OD, which was not possible at room temperature.

Supplementary Note 6

Analysis of HRTEM of PffBT4T-2OD and PffBT4T-2OD + BHT. To confirm that the 2.78 1/nm ring in the FFTs arises from π-π stacking, we compare FFTs of areas on and off (100) crystals in HRTEM of PffBT4T-2OD + BHT. **Supplementary Fig. 6a** shows the regions of interest corresponding to the edge-on, face-on, and both edge-on and face-on areas discussed in the main text for Figs. 3c, 3d, and 3e, respectively. To better understand the effect of BHT, we also compare HRTEM images and FFTs of PffBT4T-2OD and PffBT4T-2OD + BHT as a function of dose. We observe that with the addition of BHT, both (100) spacings and π-π stacking are preserved.

Supplementary Note 7

Comparison of cryogenic imaging vs room temperature imaging. We performed HRTEM of PffBT4T-2OD at cryogenic conditions as a benchmark for imaging at room temperature with the addition of antioxidants. As seen in Fig. 4 in the main text, cryogenic TEM revealed the same π-π stacking morphology that was observed at room temperature with the addition of BHT. We compare the image at cryogenic conditions with the one at room temperature using the radial

integrations of their Fast Fourier Transforms (FFTs). We see that in both images, the π stacking peak occurs at around q = 1.72 1/Å, which corresponds to 3.6 Å. Using the moving average (**Supplementary Fig. 7**, shown in black) as a baseline, we calculate signal-to-noise by dividing the height of the peak by the average deviation from the baseline. The signal-to-noise of the image taken at cryogenic conditions is more than four times higher than the signal-to-noise in the image taken at room temperature.

Cryogenic imaging experiments were conducted on a Falcon 3ec detector, whereas the room temperature imaging experiments were conducted on a K2 detector. The DQE of the Falcon 3ec is higher than that of the K2; for example, at 0.5 fraction of the Nyquist limit, the DQE of the Falcon 3ec is ~0.8 whereas for the K2 it is ~0.5.[3, 4] The superior signal in the image taken at cryogenic conditions is thus partially due to the better DQE, although the higher dose enabled by cryogenic conditions also plays a role. Nevertheless, we observe similar morphologies at cryogenic conditions compared to at room temperature with BHT, demonstrating that BHT can enable high resolution studies at room temperature.

Supplementary Note 8

Schematic of beam damage without and with antioxidant. We hypothesize that as the beam passes through the sample, it generates a free radical reacting species in the conjugated polymers, likely from scission of the side chains. These free radicals can then diffuse around, causing further damage to the material. In the presence of an antioxidant, however, the reacting species can be quenched by the antioxidant before causing further damage (**Supplementary Fig. 8**).

Supplementary Note 9

Mechanisms for quenching of reacting species by antioxidants. Proposed mechanisms of BHT and TEMPO interacting with an alkyl radical are shown in **Supplementary Fig. 9**. BHT is likely more effective at stabilizing polymers under the electron beam because the BHT radical is more stable than the nitroxide radical due to its resonance and steric hinderance.

Supplementary Note 10

Optimal concentration of antioxidant. A concentration of 7 wt% antioxidant was used in this study. This concentration was chosen because previous literature using hindered phenol antioxidants to reduce UV-radiation damage in organic photovoltaics found that < 10 wt% provided UV radiation protection without decreasing percent conversion efficiency while 20 wt% would begin to decrease percent conversion efficiency in the organic photovoltaic for most of the tested additives.[5] Using P3HT and BHT, we tested a range of concentrations from 4 wt% to 20 wt% and found that the advantageous effects of BHT are maximized at 7 wt%. The ratio of the critical dose with and without BHT, $D_{C_{BHT}}/D_{C_{neat}}$, is 1.7 for 7 wt% in the main text, but a different batch of P3HT and 7.4 wt% BHT was used for the investigation of the effect of concentration of BHT, where $D_{C_{BHT}}/D_{C_{neat}} = 1.5$ (P3HT M_w = 50.9 kg/mol for results shown in main text and P3HT M_w = 29.5 kg/mol for **Supplementary Fig. 10**). $D_{C_{neat}}$ of P3HT with M_w = 29.5 kg/mol is 12.9 e/Å^2 and with M_w = 50.9 kg/mol it is 10.4 e/Å^2. The increase in $D_{C_{neat}}$ with lower molecular weight may be due to higher crystallinity, because crystalline phases are likely more resistant to radiation damage.

Supplementary References

1. Kumar S, Adams WW. Electron beam damage in high temperature polymers. *Polymer* **31**, 15-19 (1990).

2. Grubb DT. Radiation damage and electron microscopy of organic polymers. *Journal of Materials Science* **9**, 1715-1736 (1974).

3. Falcon 3EC Direct Electron Detector Datasheet. Thermo Scientific.

4. McMullan G, Faruqi AR, Clare D, Henderson R. Comparison of optimal performance at 300 keV of three direct electron detectors for use in low dose electron microscopy. *Ultramicroscopy* **147**, 156-163 (2014).

5. Turkovic V, Engmann S, Tsierkezos N, Hoppe H, Ritter U, Gobsch G. Long-term stabilization of organic solar cells using hindered phenols as additives. *ACS Appl Mater Interfaces* **6**, 18525-18537 (2014).
